# Perception-action coupling during discriminative interceptive actions

**DOI:** 10.3389/fpsyg.2025.1526153

**Published:** 2025-04-30

**Authors:** Yu Sun, Dukchan Jang, Sangbum Park

**Affiliations:** ^1^Department of Physical Education, Yancheng Teachers University, Yancheng, China; ^2^Department of Physical Education, Keimyung University, Daegu, Republic of Korea

**Keywords:** interceptive action, perception-action coupling, spatiotemporal accuracy, eye-hand coordination, discriminative task

## Abstract

**Introduction:**

Interception is a complex task that requires the integration of perception and action under temporal constraints. Decision-making about whether to respond to moving stimuli involved in discriminative responses may further increase the cognitive load imposed on the performer, influencing perception-action coupling during interception. This study investigated the effects of discriminative response requirements on eye and hand movements, the coupling of perception and action, and the accuracy of responses during interceptive actions.

**Methods:**

Twelve right-handed male participants performed interceptive actions to stimuli moving at three velocities (0.53 m/s, 0.66 m/s, 0.88 m/s) in discriminative (target-specific) and non-discriminative (target non-specific) conditions. While the non-discriminative condition required participants to respond to presented stimuli in all trials, the discriminative condition required them to respond to the stimulus moving toward a pre-defined target area.

**Results:**

Timing errors were greater in the discriminative condition than the non-discriminative condition, and increased with increasing stimulus velocity. Both reaction and movement times decreased with increasing stimulus velocity, and the reaction times were longer in the discriminative condition than the non-discriminative condition. Variables representing the temporal aspects of interceptive actions, including saccadic latency, saccadic frequency, gaze duration, and temporal coupling of gaze and stimulus decreased with increasing stimulus velocity. Compared to the non-discriminative condition, saccadic frequency was higher, gaze duration was shorter, and the temporal coupling of gaze and stimulus was longer in the discriminative condition. Variables representing the spatial aspects of responses, including radial error, gaze error, and the spatial couplings of gaze and hand, however, remained unaffected by task conditions.

**Conclusion:**

These findings suggest that decision-making about whether to respond to moving stimuli may impair the temporal accuracy of responses by delaying perception-action coupling without severe influences on the spatial coupling of eye and hand during interceptive actions.

## 1 Introduction

Anticipation timing ability plays an important role during interceptive actions, requiring responses that coincide with the time and location that moving objects arrive at a pre-defined target area (Harrold and Kozar, 2002; Marinovic et al., 2008; Park, 2020; Rothenberg-Cunningham and Newell, 2013). Control of interceptive actions is comprised of the perception of object trajectory and the planning and execution of a required response (Lee and Park, 2023; Lim, 2015; Park, 2020; Warren, 2006; Williams, 2000). To produce accurate interceptive actions, a performer requires perception of object trajectory and execution of eye and hand movements toward the predicted location of object arrival at the pre-defined area (Park, 2020; Williams, 2000). Prediction of the time and location of object arrival is based on visual perception of object velocity and direction occurring during the latency of eye movements (Fooken and Spering, 2019; Hayhoe et al., 2012). Prior to movement initiation, gaze is usually fixated at the current position of an object, and when the object moves at velocities over 30°/s, saccadic eye movements occur to move the gaze toward the direction of the moving object after saccadic latency (Bennett et al., 2007; Brenner and Smeets, 2011; Lee and Park, 2023; Park, 2003, 2005). The stimulus and direction of moving objects perceived during the saccadic latency are used in planning eye and hand movements occurring during the response, determining the time and location of gaze fixation at the target area (Hayhoe et al., 2012; Kishita et al., 2020; Tresilian and Plooy, 2006; Williams, 2000). The spatiotemporal accuracy of interceptive actions is determined by the accuracy of both perception and action, which can be represented by spatiotemporal relations between target, gaze, and hand (Lappin et al., 2021; Warren, 2006).

Previous studies interested in the control of interceptive actions have found that the spatiotemporal accuracy, eye movements, and the coupling of eye and hand occurring during the response are modulated by the velocity and uncertainty of moving objects (Brenner and Smeets, 2017; Croft et al., 2010; de la Malla et al., 2017; Márquez and Treviño, 2024a; Tochikura et al., 2020; Treviño et al., 2024). When the target speed and directional uncertainty were manipulated during a visuomotor task, the onset and amount of optimal interception strategy depended on task parameters, increasing stimulus velocity and uncertainty leading to increased perceptual error in eye and hand movement during a task to chase a moving dot (Márquez and Treviño, 2024a). When chasing or escaping a dot moving at speeds from 10°/s to 60°/s, increasing target speed lowered performance of both tasks, while directional uncertainty compromised chasing performance but improved escaping (Treviño et al., 2024). Object velocity also affects eye and hand movements occurring after saccadic latency. Spatiotemporal errors and the distance between points of gaze and hand at movement termination increased with increasing stimulus velocity, while the latency and frequency of saccades and the intervals between arrival times of gaze and hand at the target area decreased with increasing stimulus velocity during interceptive actions to stimuli moving at various velocities toward different directions (Lee and Park, 2023; Park, 2020). Directional uncertainty, however, did not affect eye and hand movements and their coupling, suggesting that information about stimulus direction, as well as stimulus velocity, is perceived during the saccadic latency and incorporated in planning the point of gaze and hand at the target area (Park, 2020).

Interceptive actions are performed under considerable time pressure because they require responses in coincident with the arrival of a moving object at a target area (Fooken and Spering, 2019; Hayhoe, 2017; Owens et al., 2018). An increase in object velocity further increases the temporal constraints imposed on the response by reducing the time allowed for the visual processing of stimulus information. When an object moves toward the target area, gaze shifts in the direction of object’s trajectory after a saccadic latency and fixates on the predicted location of object arrival in advance of the hand. With increasing object velocity, perceptual errors in predicting the time and location of object arrival at the target area also increases, resulting in a decrease in the spatiotemporal accuracy of gaze and hand movements and their coupling (Deconinck et al., 2011; Etchells et al., 2010; Treviño and Márquez, 2025). These results suggest that the velocity of a moving object perceived during saccadic latency affects the time of gaze fixation at the target area and the initiation of hand movement, determining the temporal coupling of eye and hand during interceptive actions. An increase in object velocity can also affect gaze fixation at the target area, decreasing its accuracy and increasing the interval between gaze fixation and hand arrival (Nakazato et al., 2024). The decrease in the accuracy of gaze fixation at the target area with increasing object velocity suggests that perceived information about object trajectory affects both spatial and temporal accuracies of interceptive actions (Etchells et al., 2010; Park, 2020).

The pattern of eye-hand coordination observed during interceptive actions suggests that the gaze pattern determined by the perceived object trajectory is spatiotemporally coupled with the hand movement and acts as an important criterion in determining the accuracy of responses (Lee and Park, 2023; Lim, 2015; Park, 2020). Previous studies, however, have not considered how the decision-making process of whether or not to respond to moving stimuli affects perception-action coupling during interceptive actions, as they required subjects to respond to presented stimuli in all trials (Chen et al., 2021; Owens et al., 2018). In the real-world situation where interceptive actions are performed, however, a discriminative response to moving objects satisfying certain conditions is required. For example, a batter in baseball does not hit all the balls pitched but passes through a decision-making process to determine if the ball will arrive at the strike zone or not based on the perceived ball trajectory and initiates a bat swing only when he/she decides to hit the ball (Chen et al., 2021; Fooken and Spering, 2019; Owens et al., 2018). Discriminative responses to moving objects, such as in a baseball batting situation, may further increase temporal constraints already imposed on interceptive actions by demanding additional cognitive activity to make decisions about whether to respond or not (Bennur and Gold, 2011; Crapse et al., 2018; Farashahi et al., 2018; Hayhoe, 2017; Joo et al., 2016). To produce discriminative responses, the performer needs to decide whether to respond or not before initiating hand movements toward moving objects. This process increases the cognitive activities involved in control compared to non-discriminative situations, leading to an increase in the complexity of movement control (Farashahi et al., 2018; Joo et al., 2016; Shadlen and Kiani, 2013).

Thus, the control of interceptive actions can vary depending on the task constraints that specify how to perform the action, as well as the properties of moving objects, such as their velocities and directions. Discriminative response requirements, where a choice is needed, may affect the coupling of perception and action differently than non-discriminative situations, where a response is mandatory (Chen et al., 2021; Fooken and Spering, 2019; Hayhoe, 2017; Kim et al., 2005; Owens et al., 2018). Differences in the control between discriminative and non-discriminative interceptive actions can be reflected in the eye movements, spatiotemporal couplings among stimulus, gaze, and action, and the accuracy of response. This study investigated the effect of discriminative response requirements on eye and hand movements, the coupling of perception and action, and the accuracy of response during interceptive actions, and the results would contribute to a deeper understanding of control mechanisms for interceptive actions.

## 2 Methods

### 2.1 Participants

Twelve healthy male university students (*M*age = 23.4, *SD* = 2.4) participated in this study. All participants were right-handed and had normal or corrected-to-normal vision. They were assessed for their physical and mental states prior to the experiment, and all reported being in good health, with no recent cognitive or neurological issues. Participants voluntarily read and signed an informed consent form before the experiment. The study protocol was in accordance with the Declaration of Helsinki and was approved by the ethics committee of the Institutional Review Board of Keimyung University, South Korea: IRB No. 40525-202211-HR-069-04.

### 2.2 Experimental setup

**Figure 1:**
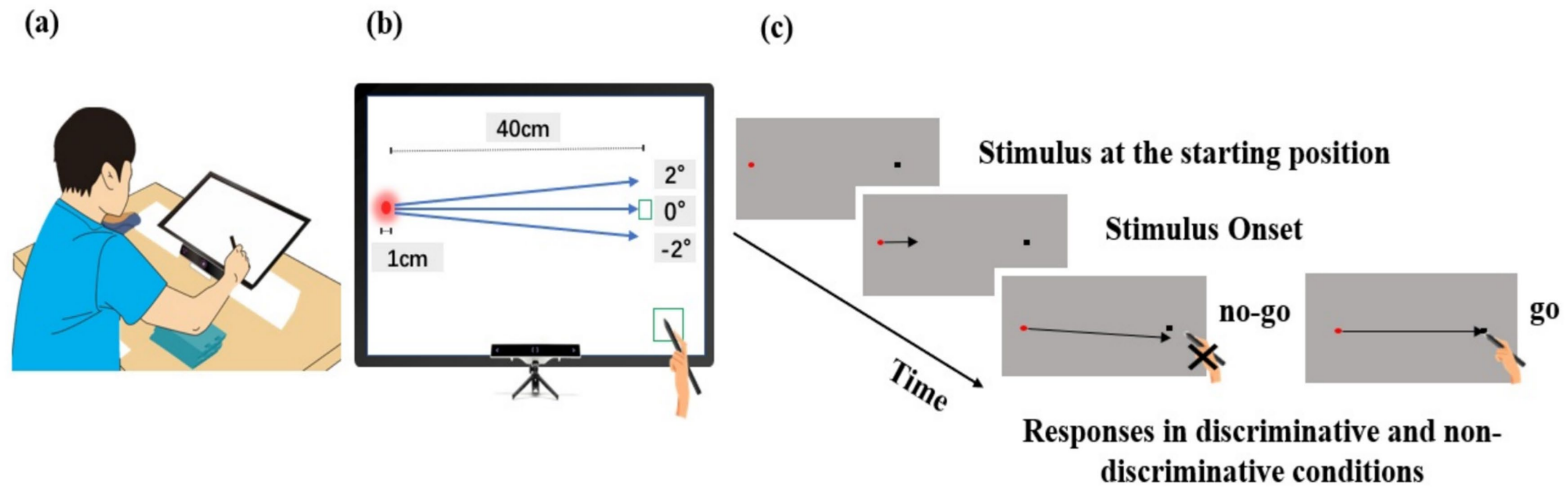
Experimental setup. **(a)** Participants were seated in a height-adjustable chair with their chin placed on a chin rest, positioned 30 cm away from the touchscreen. **(b)** Eye movements were tracked using an eye-tracker mounted beneath the touchscreen monitor. The touchscreen monitor was used to present stimuli moving at various velocities in three directions toward the predefined target area. It allowed for the measurement of eye and hand movements synchronously as required in the experimental design. **(c)** For each trial, participants were required to contact the stylus on the preparation area and shift their gaze to the starting position of the stimulus. Approximately 2 to 3 s after the stylus contacted the preparation area, the stimulus began to move toward the target area, located 40 cm to the right of the starting position. In the non-discriminative condition, the stimulus moved toward the 0° direction at one of three velocities, and participants were required to hit the stimulus coincident with its arrival at the target area. In the discriminative condition, the stimulus moved toward the target area at one of three velocities in one of three directions, and participants were required to hit the stimulus only if it moved toward the 0° direction relative to the predefined target area.

Figure 1 illustrates the experimental setup. A touchscreen monitor (U24OLED Edge HDMI, BitM Inc., Korea) was used to present a circular stimulus of 1 cm in diameter moving at various velocities in three directions. A chin rest was used to prevent head movement during responses, and a stylus (CS140B, Wacom Co., Japan) was used to perform interactive actions. Participants were required to hit the moving stimulus coincident with its arrival at a pre-defined target area located 40 cm rightward from its starting position. From the subject’s point of view, the angular distance between the starting position of the stimulus and the target area was about 35°. Data collection began when participants touched a preparation area with the stylus and ended upon response completion. For each response, timing errors were measured in milliseconds, and spatial errors and eye movements were recorded as *x* and *y* coordinates in millimeters by digital conversion. Eye movements were tracked using an eye-tracker (GP3HD, Gazepoint, Inc., Vancouver, Canada) mounted beneath the touchscreen monitor at a sampling rate of 150 Hz with a sampling error from 0.5 to 1°. The use of the touchscreen monitor allowed for the presentation of stimuli in various directions toward a target area and the measurement of eye and hand movements synchronously as required in this experimental design. Although there was a concern regarding potential processing delay in participants’ responses due to the use of the touchscreen monitor, this effect seemed to be minimal since the response time of the capacitive monitor used in this study was less than 6 milliseconds, and this delay was applied to all participants.

### 2.3 Experimental procedure

Participants were tested individually in a controlled setting. They were seated in a height-adjustable chair with their chin placed on a chin rest, positioned 30 cm away from the touchscreen. Before collecting eye movement data, the eye-tracker was calibrated by having participants shift their gaze to nine dots displayed on the touchscreen monitor. After the eye tracker correctly detected the pupil, participants were provided with a clear explanation of the task and instructions on how to perform it. Participants performed interceptive actions in two task conditions that differed in their response requirements. In a non-discriminative condition, participants were required to perform interceptive actions in response to presented stimuli in every trial. In contrast, a discriminative condition required responses only in trials where the stimulus was moving toward a pre-defined target area.

Before collecting experimental data for each task condition, participants underwent a practice session to familiarize themselves with the test procedure. In the non-discriminative condition, 10 practice trials were provided. For each trial, participants were required to contact the stylus on the preparation area and shift their gaze to the starting position of the stimulus without head or body movements, upon hearing a verbal ‘ready’ signal. Approximately 2 to 3 s after the stylus contacted the preparation area, the stimulus began to move toward the center of the target area, located 40 cm to the right of the starting position, at a velocity of 0.6 m/s. Participants were required to hit the stimulus coincident with its arrival at the target area. The test procedure for the practice session in the discriminative condition was similar to that in the non-discriminative condition, except for the stimulus trajectories and response requirements. In the discriminative condition, the stimulus could move toward the target area in one of three directions: 2°, 0°, and −2° relative to the pre-defined target area. Participants were required to hit the stimulus only when it moved toward the 0° direction. Three directions of stimuli were presented 10 times each in a random order. After each practice trial, feedback about spatiotemporal errors of response and instruction about how to perform the task were provided to help participants better understand the task instructions.

With the completion of the practice session in each task condition, participants performed experimental trials. While the test procedure and task requirements remained the same as in the practice session, stimulus velocities and directions differed by task conditions. In the non-discriminative condition, the stimulus moved toward the 0° direction at one of three velocities (0.53 m/s, 0.66 m/s, and 0.88 m/s), and participants were required to hit the stimulus coincident with its arrival at the target area, both in time and location. In the discriminative condition, the stimulus moved toward the target area at one of three velocities in one of three directions: 2°, 0°, and −2° relative to the pre-defined target area. Participants were required to hit the stimulus only if it moved toward the 0° direction. The presentation order of the stimulus velocities and directions was randomized by trials using an MS Excel program (Microsoft Corporation, Redmond, WA), and participants were unaware of the order of the manipulations.

Durations from stimulus onset to its arrival at the target area were determined by the stimulus velocity and the distance between the starting position of the stimulus to the pre-defined target area, and ranged from 450 ms (0.88 m/s) to 750 ms (0.53 m/s). Since the stimulus moved linearly from the starting position to the target area at a constant speed, the m/s unit was used in specifying the stimulus velocity. Participants performed a total of 60 trials in the non-discriminative condition, with 20 trials for each stimulus velocity, and 180 trials in the discriminative condition, with 20 trials for each combination of three stimulus velocities and three stimulus directions. The presentation order of task conditions was randomly assigned to participants, and a 5-min rest was provided after the completion of experimental trials in each task condition. During the experimental session, knowledge of results on the spatial and temporal accuracy of responses was not provided to avoid undue influences on the control strategy of participants. Task instructions to hit the stimulus coincident with its arrival at the target area were provided every 10 trials to keep participants motivated throughout the experimental session.

### 2.4 Data analysis

Data collected in each task condition were used to calculate variables representing the spatiotemporal accuracy and response speed of interceptive actions, eye movement patterns occurring during responses, and the coupling of eye and hand at movement endpoint. In the discriminative condition, data collected from only the 0° direction were used to calculate these variables.

#### 2.4.1 Spatiotemporal accuracy and response speed

Timing error (ms) was determined by the absolute time difference between the arrival of the stimulus at the target area and the contact of the stylus on the touchscreen. Radial error (mm) was defined as the interval between the location of stimulus arrival at the target area and the contact point of the stylus at the end of the response, calculated as follows:


$$\text{Radial error = }\sqrt{\left(x^{2}+y^{2}\right)}\text{,}$$

where x represents the interval between the location of stimulus arrival and the contact point of the stylus on the x axis, and y represents the interval between the location of stimulus arrival and the contact point of the stylus on the y axis. Reaction time (ms) was defined as the time interval from the onset of the stimulus to the lifting of the stylus from the preparation area. Movement time (ms) represented the time interval from the lifting of the stylus to the completion of the response.

#### 2.4.2 Eye movement patterns

Saccadic latency (ms) was determined by the time from the onset of the stimulus to the initiation of saccades, and saccadic frequency represented the number of saccades occurring in each response. Gaze duration (ms) referred to the duration of gaze fixation at the target area before stimulus arrival, calculated by subtracting the time of gaze arrival at the target area from the time of stimulus arrival. Gaze error (mm) was defined as the interval between the location of stimulus arrival at the target area and the point of gaze at the end of the response, calculated as follows:


$$\text{Gaze error = }\sqrt{\left(x^{2}+y^{2}\right)}\text{,}$$

where x represents the interval between the location of stimulus arrival and the point of gaze on the x axis, and y represents the interval between the location of stimulus arrival and the point of gaze on the y axis.

#### 2.4.3 Eye-hand coupling

Temporal coupling (ms) represented the temporal relationship between eye and hand movements. It was calculated by subtracting the time of gaze arrival at the target area from the time of response completion. Spatial coupling (mm) represented the spatial relationship between eye and hand movements, and was determined by the interval between the point of gaze and the contact point of the stylus at response completion. Spatial coupling was calculated as follows:


$$\text{Spatial coupling = }\sqrt{\left(x^{2}+y^{2}\right)}\text{,}$$

where x represents the interval between the point of gaze and the contact point of the stylus on the x axis, and y represents the interval between the point of gaze and the contact point of the stylus on the y axis.

Calculated variables were analyzed by two-way ANOVAs with repeated measures, using task conditions (discriminative, non-discriminative) and stimulus velocities (0.53 m/s, 0.66 m/s, 0.88 m/s) as factors. In all analyses using repeated measures ANOVA, the Greenhouse–Geisser correction was applied to correct for non-sphericity when necessary. For pairwise comparisons, Bonferroni’s *post hoc* test was used. The significance level for all analyses was set at *p* < 0.05. Data were analyzed with IBM SPSS Statistics Version 26 and are presented as means and standard errors.

## 3 Results

### 3.1 Spatiotemporal accuracy and response speed

#### 3.1.1 Timing error

**Figure 2:**
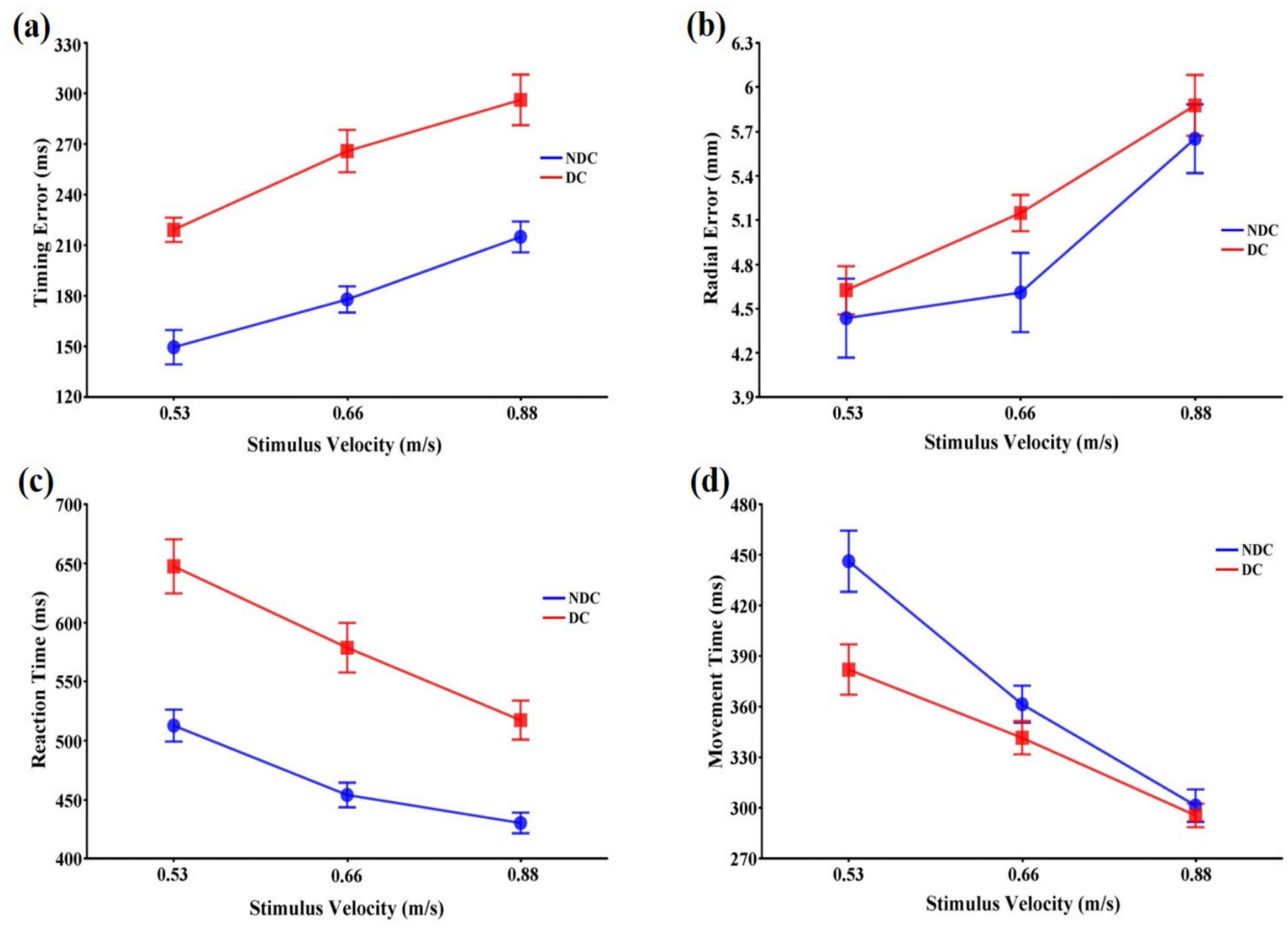
Means and standard errors of variables representing the spatiotemporal accuracy and response speed of interceptive actions in discriminative and non-discriminative conditions. **(a)** Time error (ms); **(b)** Radial error (mm); **(c)** Reaction time (ms); **(d)** Movement time (ms). Error bars represent standard errors of the means. DC: Discriminative condition, NDC: Non-discriminative condition.

Analysis of timing error showed significant main effects for both task condition, *F*(1,11) = 88.78, *p* < 0.001, $\eta_{p}^{2}$ = 0.890, and stimulus velocity, *F*(2,22) = 67.57, *p* < 0.001, $\eta_{p}^{2}$ = 0.860. The timing error was significantly larger in the discriminative condition than in the non-discriminative condition (*p* < 0.001), and increased with increasing stimulus velocity (*p* < 0.01). The interaction between the task condition and the stimulus velocity was not significant, *F*(2,22) = 0.90, *p* > 0.05, $\eta_{p}^{2}$ = 0.076 (Figure 2a).

#### 3.1.2 Radial error

The stimulus velocity also had a significant main effect on radial error, *F*(2,22) = 15.98, *p* < 0.001, $\eta_{p}^{2}$ = 0.592, which increased with increasing stimulus velocity (*p* < 0.05). The main effect of the task condition, *F*(1,11) = 1.34, *p* > 0.05, $\eta_{p}^{2}$ = 0.109, and the interaction between the task condition and the stimulus velocity, *F*(2,22) = 2.56, *p* > 0.05, $\eta_{p}^{2}$ = 0.189, were not significant (Figure 2b).

#### 3.1.3 Reaction time

Both task condition, *F*(1,11) = 27.10, *p* < 0.001, $\eta_{p}^{2}$ = 0.711, and stimulus velocity, *F*(2,22) = 63.65, *p* < 0.001, $\eta_{p}^{2}$ = 0.853, had significant main effects on reaction time. The interaction between them was also significant, *F*(2,22) = 10.91, *p* < 0.01, $\eta_{p}^{2}$ = 0.498, implying that differences in the reaction time between task conditions might vary depending on the stimulus velocity (Figure 2c). Subsequent tests revealed that while the reaction time increased with increasing stimulus velocity and was longer in the discriminative condition than in the non-discriminative condition (*p* < 0.01) at all stimulus velocities, differences in the reaction time between task conditions was larger in the 0.53 m/s and 0.66 m/s velocity conditions than in the 0.88 m/s velocity condition (*p* < 0.001).

#### 3.1.4 Movement time

While the task condition did not affect movement time, *F*(1,11) = 7.18, *p* > 0.05, $\eta_{p}^{2}$ = 0.395, the stimulus velocity had a significant main effect on the movement time, *F*(2,22) = 55.54, *p* < 0.001, $\eta_{p}^{2}$ = 0.835. The interaction between these two variables was also significant, *F*(2,22) = 5.96, *p* < 0.01, $\eta_{p}^{2}$ = 0.352, indicating differences in the movement time between task conditions might vary depending on the stimulus velocities (Figure 2d). Subsequent tests for the interaction revealed that while the movement time in the 0.53 m/s velocity condition was longer in the non-discriminative condition than in the discriminative condition (*p* < 0.05), those in the 0.66 m/s and 0.88 m/s velocity conditions were not significantly different between task conditions (*p* < 0.05). While the movement time decreased with increasing stimulus velocity in the non-discriminative condition (*p* < 0.01), the movement time in the 0.88 m/s velocity condition was significantly shorter than those in the 0.66 m/s and 0.53 m/s velocity conditions (*p* < 0.01) in the discriminative condition.

### 3.2 Eye movement patterns

#### 3.2.1 Saccadic latency

**Figure 3:**
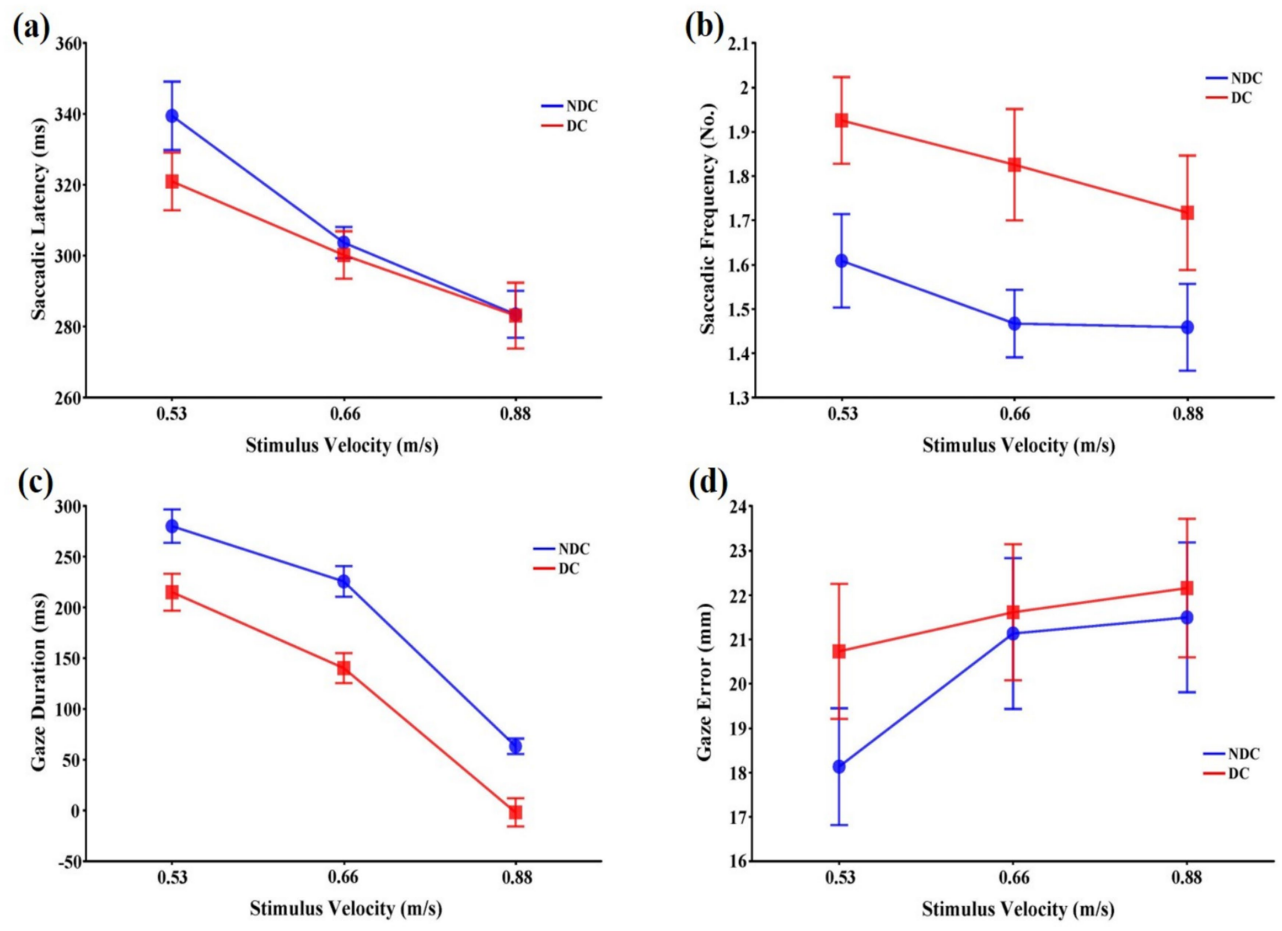
Means and standard errors of variables representing eye movement patterns that occur during interceptive actions in discriminative and non-discriminative conditions. **(a)** Saccadic latency (ms); **(b)** Saccadic frequency (No.); **(c)** Gaze duration (ms); **(d)** Gaze error (mm). Error bars represent standard errors of the means. DC: Discriminative condition, NDC: Non-discriminative condition.

The stimulus velocity affected saccadic latency, *F*(2,22) = 27.77, *p* < 0.001, $\eta_{p}^{2}$ = 0.716, which decreased with increasing stimulus velocity (*p* < 0.01). However, the main effect of the task condition, *F*(1,11) = 0.51, *p* > 0.05, $\eta_{p}^{2}$ = 0.044, and the interaction between the task condition and the stimulus velocity, *F*(2,22) = 2.64, *p* > 0.05, $\eta_{p}^{2}$ = 0.194, were not significant (Figure 3a).

#### 3.2.2 Saccadic frequency

Both task condition, *F*(1,11) = 22.00, *p* < 0.01, $\eta_{p}^{2}$ = 0.667, and stimulus velocity, *F*(2,22) = 5.32, *p* < 0.05, $\eta_{p}^{2}$ = 0.326, had significant main effects on saccadic frequency (Figure 3b). Post-hoc tests revealed that the saccadic frequency was significantly higher in the discriminative condition than in the non-discriminative condition (*p* < 0.01), and in the 0.53 m/s velocity condition than in the 0.66 m/s and 0.88 m/s velocity conditions (*p* < 0.05), which were not significantly different from each other (*p* > 0.05). The interaction between the task condition and the stimulus velocity was not significant, *F*(2,22) = 0.69, *p* > 0.05, $\eta_{p}^{2}$ = 0.059.

#### 3.2.3 Gaze duration

The main effects of the task condition, *F*(1,11) = 53.56, *p* < 0.001, $\eta_{p}^{2}$ = 0.830, and the stimulus velocity, *F*(2,22) = 163.33, *p* < 0.001, $\eta_{p}^{2}$ = 0.937, on gaze duration were also significant (Figure 3c). Post-hoc tests revealed that the gaze duration was significantly longer in the non-discriminative condition than in the discriminative condition (*p* < 0.01), and decreased with increasing stimulus velocity (*p* < 0.001). However, the interaction between the task condition and the stimulus velocity was not significant, *F*(2,22) = 0.78, *p* > 0.05, $\eta_{p}^{2}$ = 0.066.

#### 3.2.4 Gaze error

The stimulus velocity had a significant main effect on gaze error, *F*(2,22) = 16.75, *p* < 0.001, $\eta_{p}^{2}$ = 0.604, which was smaller in the 0.53 m/s velocity condition than the 0.66 m/s and 0.88 m/s velocity conditions (*p* < 0.05). However, the main effect of the task condition, *F*(1,11) = 0.67, *p* > 0.05, $\eta_{p}^{2}$ = 0.058, and the interaction between the task condition and the stimulus velocity, *F*(2,22) = 2.84, *p* > 0.05, $\eta_{p}^{2}$ = 0.206, were not significant (Figure 3d).

### 3.3 Eye-hand coupling

#### 3.3.1 Temporal coupling

**Figure 4:**
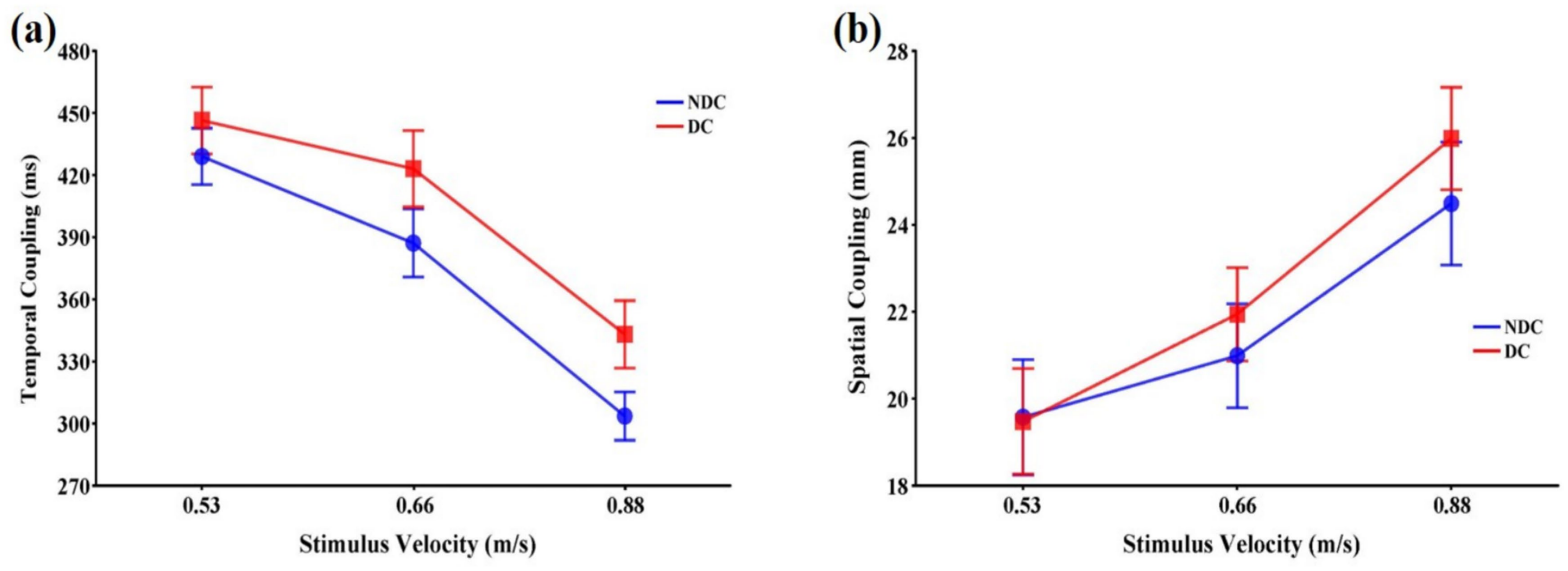
Means and standard errors of variables representing the coupling of eye and hand that occurs during interceptive actions in discriminative and non-discriminative conditions. **(a)** Temporal coupling (ms); **(b)** Spatial coupling (mm). Error bars represent standard errors of the means. DC: Discriminative condition, NDC: Non-discriminative condition.

The task condition, *F*(1,11) = 9.26, *p* < 0.05, $\eta_{p}^{2}$ = 0.457, and the stimulus velocity, *F*(2,22) = 94.26, *p* < 0.001, $\eta_{p}^{2}$ = 0.896, had significant main effects on temporal coupling (Figure 4a). Post-hoc tests revealed that the temporal coupling was significantly longer in the discriminative condition than in the non-discriminative condition (*p* < 0.05), and became shorter with increasing stimulus velocity (*p* < 0.05). The interaction between the task condition and the stimulus velocity was not significant, *F*(2,22) = 0.85, *p* > 0.05, $\eta_{p}^{2}$ = 0.072.

#### 3.3.2 Spatial coupling

The stimulus velocity had a significant main effect on spatial coupling, *F*(2,22) = 19.29, *p* < 0.001, $\eta_{p}^{2}$ = 0.637, which became larger with increasing stimulus velocity. However, the main effect of the task condition, *F*(1,11) = 0.78, *p* > 0.05, $\eta_{p}^{2}$ = 0.067, and the interaction between the task condition and the stimulus velocity, *F*(2,22) = 0.29, *p* > 0.05, $\eta_{p}^{2}$ = 0.026, were not significant (Figure 4b).

## 4 Discussion

Discriminative responses to a stimulus satisfying specific conditions increase the uncertainty imposed on interactive actions. The performer needs to make decisions about whether to respond or not to resolve this uncertainty before initiating hand movements (Fooken and Spering, 2019; Hayhoe, 2017; Márquez and Treviño, 2024b; Owens et al., 2018). However, increased uncertainty due to discriminative response requirements does not seem to affect the interception strategies of participants. When a stimulus moves at a constant speed and in a predictable trajectory, predictive interception strategies are selected to anticipate the future location of the stimulus by considering its position, trajectory, and speed (Márquez and Treviño, 2024c). When the trajectory of a stimulus is unpredictable, however, prospective strategies involving continuous monitoring of the stimulus trajectory and real-time movement adjustments are selected to intercept a target moving toward unpredictable directions (Dessing et al., 2009; Márquez and Treviño, 2024c; Merchant et al., 2009). Since the stimulus always moved from the starting position to the pre-defined target area in this experimental design, its trajectory was predictable, and participants basically employed predictive strategies to perform the tasks. Although the discriminative condition introduced uncertainty about whether to respond to moving stimuli, this increased uncertainty might not lead to the selection of different interception strategies because the predictability of the stimulus trajectories remained similar compared to the non-discriminative condition. Nevertheless, the results of this study suggest that the processing of uncertainty can affect perception-action coupling and the spatiotemporal accuracy of interactive actions by increasing cognitive load (Márquez and Treviño, 2024b).

The timing error observed in this study was larger in the discriminative condition than in the non-discriminative condition and increased with increasing stimulus velocities in both conditions. During interceptive actions, an increase in stimulus velocity may increase the temporal constraints imposed on perception-action coupling by reducing the time allowed for processing visual information about the stimulus trajectory (Kim et al., 2013). Discriminative response requirements may exacerbate the temporal constraints already imposed on the control process by increasing the cognitive activities involved in the response compared to a situation where responses to all stimuli are required (Farashahi et al., 2018). The reaction time from the onset of the stimulus to the initiation of hand movement also increased in the discriminative condition compared to the non-discriminative condition, suggesting that decision-making about whether to respond to the presented stimulus delays the planning and execution of interceptive actions. In the non-discriminative condition, hand movement can be initiated after perceiving the initial stimulus trajectory. In the discriminative condition, however, the hand can start to move after the decision to respond is made, resulting in an increase in reaction time (Danek and Mordkoff, 2011; Miller and Low, 2001). The movement time from the onset of hand movement to the completion of the response, however, was not different between the discriminative conditions, except for the 0.53 m/s velocity condition, and decreased with increasing stimulus velocities. The movement time in the 0.53 m/s velocity condition was shorter in the discriminative condition than in the non-discriminative condition, suggesting that a decrease in movement time in a slower velocity condition may compensate for the delay in reaction time due to discriminative response requirements (Marinovic et al., 2004; Nasu et al., 2020; Tresilian and Plooy, 2006). However, the movement time was not different between task conditions in the 0.66 m/s and 0.88 m/s velocity conditions, being unable to compensate for the delay in reaction time. As a result, the timing error became larger in the discriminative condition than in the non-discriminative condition, implying that the resolution of uncertainty about whether to respond or not may decrease the temporal accuracy of interceptive actions by delaying the planning and execution of hand movements (Márquez and Treviño, 2024b).

Eye movement patterns observed in the task conditions provide more direct information about the effect of uncertainty and stimulus velocity on perception-action coupling. The stimulus velocity affected the temporal aspects of eye movements and perception-action coupling. The latency of saccades ranged from 283 ms to 339 ms and increased as stimulus velocities decreased. Since visual information about the velocity and direction of a moving stimulus is perceived during saccadic latency, with the gaze fixated on the initial position of the stimulus (Sailer et al., 2003), these results suggest that increasing stimulus velocity affects the perception of the stimulus trajectory by reducing the time available for processing stimulus information (Bennett et al., 2007; Bennett et al., 2010; Croft et al., 2010; Eggert et al., 2005a; Eggert et al., 2005b; Mann et al., 2013). However, discriminative response requirements did not affect the latency of saccades, implying that the decision about whether to respond or not is made after the saccadic latency (Kveraga et al., 2002; Kveraga and Hughes, 2005).

The frequency of saccades also increased with decreasing stimulus velocities. Information about the stimulus trajectory is used to plan gaze patterns that occur after the latency of saccades during interceptive actions (Croft et al., 2010). The velocity of saccades during interceptive actions should match that of the stimulus and increase with increasing stimulus velocity (Croft et al., 2010). When the stimulus velocity is relatively slow, both smooth pursuit and saccades occur to align the point of gaze with the moving stimulus (Nakazato et al., 2024). As the stimulus velocity becomes faster, saccades occur predominantly to realign the gaze with the moving stimulus (Park, 2003, 2020). Thus, the decrease in the frequency of saccades with increasing stimulus velocity seems to reflect the process for controlling the time that the gaze reaches the target area (Eggert et al., 2005a; Eggert et al., 2005b).

The duration of gaze fixation at the target area before stimulus arrival decreased with increasing stimulus velocity due to a reduction in the duration of stimulus presentation (Lim, 2015). Nevertheless, the gaze arrived at the target area in advance of the stimulus in all velocity conditions, implying that the gaze has to be fixated at the anticipated location of stimulus arrival before the termination of hand movement, as in the aiming movements to a stationary target (Cámara et al., 2018; Mann et al., 2013). The time from gaze fixation to stylus arrival at the target area, representing the temporal coupling of eye and hand, also decreased with increasing stimulus velocity. When the distance the stimulus travels is fixed, the duration of stimulus presentation decreases as stimulus velocity increases. To produce interactive actions within a shorter time, the hand should start to move earlier and faster as the stimulus velocity increases. Thus, the temporal coupling of eye and hand observed in this study seems to reflect a process for satisfying the temporal constraints imposed by the stimulus velocities (Park, 2020).

Discriminative response requirements also affected the frequency of saccades, the duration of gaze fixation, and the temporal coupling of eye and hand, suggesting that the decision-making to resolve the uncertainty about whether to respond to moving stimuli may affect the temporal aspects of interceptive actions (Fooken and Spering, 2019; Márquez and Treviño, 2024b). The frequency of saccades was higher in the discriminative condition than in the non-discriminative condition, resulting in a reduction in eye movement velocity and a delay in the time of gaze arrival at the target area. The time taken for the gaze to arrive at the target area was delayed about 65 ms to 85 ms in the discriminative condition compared to the non-discriminative condition, resulting in a shorter duration of gaze fixation. The temporal coupling of eye and hand was also affected by task conditions, ranging from 343 ms to 446 ms in the discriminative condition and from 304 ms to 429 ms in the non-discriminative condition, respectively. These results suggest that the discriminative response requirements may weaken perception-action coupling by decreasing the velocity of eye movements and delaying the initiation of hand movements, resulting in a decrease in the temporal accuracy of interceptive actions.

Unlike the temporal aspects, the spatial aspects of interceptive actions were not affected by the discriminative response requirements. To produce interceptive actions, the performer has to anticipate the time and location of stimulus arrival at the target area based on information about the stimulus trajectory perceived during the latency of eye movements (Akl and Panchuk, 2016; Land and McLeod, 2000). Since the latency of saccades decreases with increasing stimulus velocity, the perceptual error in anticipating the time and location of stimulus arrival at the target area may increase as the stimulus velocity increases (Deconinck et al., 2011; Treviño and Márquez, 2025). In fact, the gaze error determined by the interval between the locations of stimulus arrival and gaze fixation at movement termination increased with increasing stimulus velocity. Since the location of stimulus arrival remained the same in all to-respond conditions, this result suggests that the accuracy of gaze fixation at the target area deteriorates as the stimulus velocity increases (Park, 2020). The spatial coupling of eye and hand, represented by the interval between the locations of gaze fixation and hand arrival at the target area, also increased with increasing stimulus velocity, implying that the perceptual error in anticipating the time and location of stimulus arrival at the target area may lead to a decrease in the accuracy of gaze fixations and hand movements during interceptive actions (de la Malla et al., 2017; Fooken et al., 2021). The discriminative response requirements, however, did not affect the spatial aspects of interceptive actions, including the gaze error, the spatial coupling of eye and hand, and the radial error of hand movements. Since the interval between the locations of stimulus arrival and gaze fixation reflects errors in perceiving the velocity and direction of the moving stimulus (de la Malla et al., 2017; Fooken et al., 2021), these results suggest that decision-making to resolve the uncertainty about whether to respond to a moving stimulus may not affect the accuracy of gaze fixation and hand arrival at the target area.

Taken together, the results of this study suggest that the requirement for discriminative responses has different effects on the temporal and spatial aspects of interceptive actions. While delaying the initiation of hand movement and the time of gaze fixation and hand arrival at the target area, the discriminative response requirements did not affect the gaze error and the spatial coupling of eye and hand at movement termination. These results imply that while the uncertainty involved in discriminative responses to moving stimuli may decrease the temporal accuracy of interceptive actions by delaying the coupling of perception and action, it does not affect the perception of the anticipated location of stimulus arrival and the spatial coupling of eye and hand.

While offering insights into the control mechanisms of interceptive actions in real-world situations, this study has several limitations that should be highlighted. Firstly, the small sample size of this study limits the generalizability of the findings. This study employed a repeated measures design and conducted a power analysis to determine the appropriate sample size. The power analysis using G*power with a desired power of 0.95, alpha of 0.05, estimated effect size of 0.40, and the number of repeated measures of 6 (three stimulus velocities and two task conditions) suggested a total sample size of 12. Since the repeated measures design reduces inter-individual variability and increases statistical power compared to a between-subject design, it allows for a test of the effect of independent variables with a smaller sample size. Due to this property of the repeated measures design, the stimulus velocity and task condition in this study displayed large effect sizes ranging from 0.326 to 0.937 when their main effects were significant. However, the inclusion of only 12 right-handed male university students raises questions about the generalizability of the results and potential gender bias, suggesting avenues for future research with a larger and more diverse participant pool. Additionally, although discriminative response requirements seem to increase the cognitive load on participants by demanding decision-making about whether to respond to a presented stimulus during interceptive actions, the interpretation of this implication should be approached with caution because the changes in cognitive load by task requirements were not measured directly in this study. Further investigations incorporating subjective ratings or physiological measures to assess changes in cognitive load would provide deeper insights into how discriminative response requirements affect cognitive load and perception-action coupling during interceptive actions. Finally, the experimental setup of this study lacks ecological validity, as the task of hitting a moving stimulus on a screen may not represent real-world interceptive actions, such as hitting or catching a ball. Future research should address this limitation by employing research settings that better approximate the real-world interception situation.

## 5 Conclusion

This study investigated the effects of discriminative task requirements on the spatiotemporal accuracy, gaze movements, and eye-hand coupling of interceptive actions. Key findings indicate that decision-making required under discriminative conditions can delay the initiation of responses and the time for the hand to reach the target area by increasing the uncertainty involved in interceptive actions. Decisions regarding whether to respond occur after the latency of eye movements, which may delay the temporal coupling of eye and hand by increasing saccadic frequency and decreasing saccadic velocity. However, discriminative response requirements do not seem to increase perceptual errors in predicting the point of stimulus arrival, since the intervals between the point of stimulus arrival and gaze fixation, the point of gaze fixation and hand arrival, and the points of stimulus and hand arrivals were not affected by the discriminative conditions. Thus, the increase in uncertainty due to discriminative response requirements may decrease the temporal accuracy of interceptive actions by increasing the cognitive load imposed on the control process without severely deteriorating spatial accuracy.

The resolution of uncertainty involved in discriminative responses increases cognitive load, influencing decision-making and perception-action coupling during interceptive actions (Márquez and Treviño, 2024b). The effect of uncertainty on perception-action coupling can vary depending on the difficulty of discrimination. To gain a better understanding of the impact of uncertainty on perception-action coupling, further research is needed to investigate how varying levels of discrimination difficulty affect eye-hand coupling and the spatiotemporal accuracy of interceptive actions. In addition to the uncertainty of task constraints, the velocity and size of the stimulus can influence the difficulty of perceiving its properties, increasing spatiotemporal constraints and task complexity. To deepen our understanding of the relationship between stimulus properties and response dynamics, further research is required to explore how varying sizes and velocities of stimuli affect perception-action coupling during interceptive actions.

## Data Availability

The original contributions presented in the study are included in the article/supplementary material, further inquiries can be directed to the corresponding author/s.
